# Rapid and Reliable Determination of Free and Total Glycerol in Biodiesel by Iodometric Titration

**DOI:** 10.1021/acsomega.5c04429

**Published:** 2025-09-12

**Authors:** Gabriel M. Viegas, Leonardo M. Barbosa, Érica Barbosa de Sousa, Cristiane Gimenes, José G. Rocha Junior

**Affiliations:** † Institute of Chemistry, Federal Rural University of Rio de Janeiro, Seropédica, RJ 23890-000, Brazil; ‡ Institute of Chemistry, Federal University of Rio de Janeiro, Rio de Janeiro, RJ 21941-909, Brazil

## Abstract

Biodiesel is a renewable fuel produced by the transesterification of vegetable oils or animal fats. Regulatory agencies set strict limits on free and total glycerol levels in biodiesel intended for commercialization, as glycerol can impair combustion efficiency, promote engine deposits, and generate acrolein—a highly toxic aldehyde—during combustion. Current regulations typically require gas chromatography with flame ionization detection (GC-FID) for glycerol determination, which involves expensive standards and instrumentation. The iodometric titrimetric methods ABNT NBR 15771 and ABNT NBR 15344, previously accepted by the National Agency of Petroleum, Natural Gas and Biofuels (ANP, Brazil), were discontinued due to operational complexity, notably the multiple extraction steps with toxic solvents and the interference of excess periodate, which often required reanalysis due to excessive titrant consumption in blank titrations. This work proposes the masking of excess periodate with molybdate and the elimination of the extraction step, overcoming the disadvantages associated with the original ABNT methods. The proposed methods exhibited linear response ranges of 107–1911 mg L^–1^ for total glycerol and 10.4–312 mg L^–1^ for free glycerol. Detection and quantification limits were 1.42 and 4.32 mg L^–1^ for free glycerol, and 5.35 and 16.2 mg L^–1^ for total glycerol, respectively. Repeatability RSD ranged from 2.4 to 8.8% for free glycerol and 3.3 to 7.2% for total glycerol. Interday reproducibility did not exceed 8.0%. Recovery assays for glycerol in biodiesel samples—commercial, soybean, and palm kernel—ranged from 90.9 to 106.0%. A comparison with the ASTM D6584 reference method across nine biodiesel samples demonstrated statistically equivalent results for both free and total glycerol, with values in accordance with international regulatory limits. The proposed methods provide a rapid, reliable, and low-cost approach for glycerol determination in biodiesel, offering valuable applicability for screening, educational purposes, and preliminary quality assessments.

## Graphical abstract

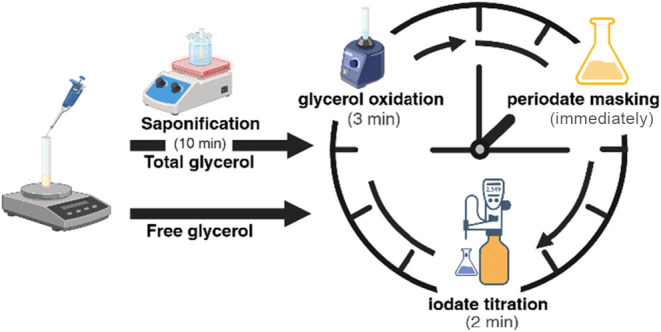

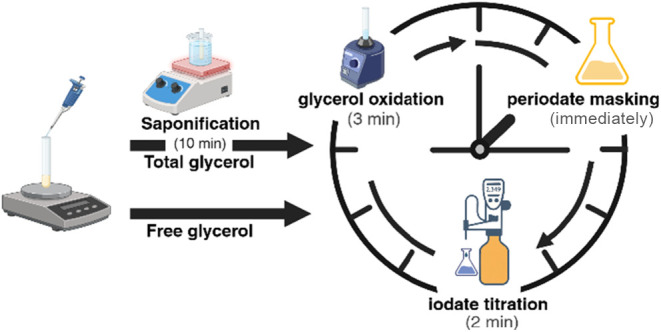

## 1 Introduction

Biodiesel is a renewable fuel and alternative to petroleum diesel, produced by the transesterification of triglycerides (from vegetable oils or animal fats) with short-chain alcohols in the presence of a catalyst.^1^ Glycerol is released as a coproduct and its presence – both as free glycerol and as part of unreacted mono-, di- and triglycerides – serves as an indicator of biodiesel quality.^2,3^

Regulatory agencies such as the National Agency of Petroleum, Natural Gas and Biofuels (ANP), the Environmental Protection Agency (EPA) and the European Committee for Standardization (CEN) established strict limits for the content of free glycerol, limiting it to 0.02% (w/w) in biodiesel. For total glycerol, the levels established by the ANP, EPA and CEN are 0.20, 0.24 and 0.25% (w/w), respectively.^4–6^ Excess glycerol can increase fuel viscosity, reduce combustion efficiency, cause injector clogging and the emission of acrolein, a toxic aldehyde.^7^

Standard methods for glycerol quantification employ gas chromatography with flame ionization detection (GC-FID), as described in ASTM D6584, EN ISO 14105 and ABNT NBR 15908. GC-FID is preferred due to its high sensitivity, accuracy, and ability to separate glycerol from other residual compounds in biodiesel. It ensures reliable detection of trace amounts and provides robust and reproducible results, which is essential for quality control and regulatory compliance.^8–10^

These methods involve derivatization with *N*-methyl-N-(trimethylsilyl)­trifluoroacetamide (MSTFA) and the use of standards, such as 1,2,4-butanetriol, ethylene glycol, 1-mono­[cis-9-octadecenoyl]-rac-glycerol, 1,2,3-tridecanolylglycerol, 1,3-di­[cis-octadecenoyl]­glycerol and 1,2,3-tri­[cis-octadecenoyl]­glycerol, among others. Many of these standards are expensive and chemically unstable, which increases both the complexity and operational cost of the analysis.^11–13^ Furthermore, the need for sophisticated instrumentation and specialized personnel limits the widespread implementation of these methods in routine laboratory settings.

Titrimetric and spectrophotometric methods have been explored as alternatives, particularly those based on the oxidation of glycerol by sodium periodate or periodic acid (Malaprade oxidation), generating measurable oxidation products such as formic acid, formaldehyde or iodate (Reaction R1).^14–28^

Reaction 1 $$C_{3}H_{8}O_{3}+2{{\mathrm{IO}}_{4}}^{-}\to 2{\mathrm{CH}}_{2}O+{\mathrm{CH}}_{2}O_{2}+2{{\mathrm{IO}}_{3}}^{-}+H_{2}O$$

The titrimetric determination of iodate produced in the Malaprade oxidation (Reaction R1) was undoubtedly the most successful strategy for quantifying glycerol, culminating in the Brazilian standards ABNT NBR 15771 and ABNT NBR 15344. These standards were officially recommended to the ANP for the determination of free and total glycerol in biodiesel, respectively.^29^ Both methods involve the extraction of glycerol from biodiesel using a biphasic mixture of an organic solvent (diethyl ether or chloroform) and acetic acid solution, followed by oxidation with periodate (Reaction R1), and subsequent iodometric titration of the iodate formed. While ABNT NBR 15771 is specific to free glycerol, ABNT NBR 15344 includes an additional initial step consisting of ester saponification to release glycerol bound to glycerides present in the biodiesel.

However, these methods have several operational limitations, including multiple analytical steps, successive macroscale analyte extractions, long reaction times (e.g., 30 min for Malaprade oxidation), and interference from unreacted periodate, which participates in the same reactions as iodate and, therefore, must be accounted for by blank titration. The high excess of periodate required for the Malaprade reaction, combined with the low concentration of glycerol typically found in biodiesel, often results in minimal differences between the volumes spent in the sample titration and the blank titration, compromising the accuracy of the quantification. To overcome this analytical constraint, ABNT NBR standards recommend reanalysis using different proportions of reagents—a practice that increases the complexity and time-consuming nature of the procedure. These disadvantages may have contributed to the recent exclusion of these methods from the list of reference standards by ANP.^4^

However, a significant methodological advance was reported by Santos et al.,^30^ who introduced the use of sodium molybdate to mask excess periodate, forming a stable complex, [I­(MoO_4_)_6_]^5–^ (Reaction R2).

Reaction 2 $${{\mathrm{IO}}_{4}}^{-}+6{{\mathrm{MoO}}_{4}}^{2-}+8H^{+}\to {\left[I{\left({\mathrm{MoO}}_{4}\right)}_{6}\right]}^{5-}+4H_{2}O$$

This innovation led to a substantial reduction in the volume of titrant consumed during blank titration, thereby enabling the reliable quantification of glycerol at low concentration (10 mg L^–1^).^30^ Furthermore, the approach allowed the use of a relatively high excess of periodate, making the Malaprade oxidation rapid and quantitative, with reaction times reduced to only 3 min. According to Santos et al.,^30^ the method demonstrated high reliability in aqueous systems and was successfully applied to the determination of free glycerol in biodiesel following analyte extraction by liquid–liquid partition. However, the application of molybdate for periodate masking was not investigated by the authors for total glycerol determination. Moreover, the results for free glycerol were not compared with those obtained using reference methods or standard samples.

The present work proposes the determination of free and total glycerol in biodiesel by means of iodometric titration of iodate generated from the oxidation of glycerol by periodate, with subsequent masking of residual periodate using molybdate. The methodologies described herein are intended to simplify the original procedure for free glycerol quantification by eliminating the analyte extraction step, while also extending its applicability to the determination of total glycerol—an approach not previously explored.

## 2 Materials and Methods

### 2.1 Materials

Biodiesel samples were kindly provided by the Laboratory of Fuels and Petroleum Derivatives (LABCOM/UFRJ), Rio de Janeiro, Brazil (samples 1–8), and by Raízen’s production unit located in Duque de Caxias, Rio de Janeiro, Brazil (commercial biodiesel). According to the supplier, this commercial biodiesel is produced from a blend of diverse raw materials, including soybean oil (31.3%), used frying oil (20.2%), pork fat (1.7%), chicken oil (3.5%), mixed vegetable oil (29.0%), mixed animal fats (11.4%), corn oil (0.2%), beef tallow (0.7%), and sunflower oil (2.0%). The product also contains *tert*-butylhydroquinone (TBHQ) at a concentration of 120 mg L^–1^, as reported by the manufacturer. Additional biodiesel samples were synthesized from soybean oil, which is rich in unsaturated fatty acids, and palm kernel oil, known for its higher degree of saturation,^31^ following the methodology described by Santos et al.^30^ This compositional variability among samples ensures a representative range of biodiesel matrices for method validation. All biodiesel samples investigated were synthesized via the methyl route.

Sodium periodate (≥99.8% w/w) was obtained from Meta, Brazil. Sodium molybdate dihydrate (≥99.8% w/w), concentrated sulfuric acid (95–98% w/w), glacial acetic acid (≥99.7% w/w), sodium hydroxide (97% w/w), potassium iodate (≥99.4% w/w), and glycerol (99.5% w/w) were purchased from Sigma-Aldrich, Brazil. Potassium iodide (99.5% w/w) was obtained from Êxodo Científica, Brazil, and starch (≥95% w/w) from Cinética Reagentes e Soluções, Brazil. Sodium thiosulfate (≥99.5% w/w) was supplied by Impex, Brazil. Potassium hydroxide (90.6% w/w) was obtained from Katrium Indústrias Químicas S.A, Brazil. Phosphoric Acid (85% w/w) was obtained from Sciavicco, Brazil. A 70% (v/v) ethanolic solution was prepared by diluting absolute ethanol (99.5% w/w, Proquímios, Brazil) with water and is hereafter referred to as ″ethanol″ for simplicity. Standard glycerol solutions were prepared by dissolving glycerol (99.5% w/w) in ethanol. All reagents were used without further purification. Solutions were prepared using deionized water.

Volumetric flasks (class A) and micropipettes with manufacturer-certified accuracy were used. Titrations were performed using a 10 mL digital buret (±0.001 mL, Brand Titertte, Germany). Analytical balances with a precision of ± 0.1 mg were used to weigh biodiesel samples and standard reagents.

### 2.2 Methods

#### 2.2.1 Standardization of Na_2_S_2_O_3_ Solutions

An aliquot of 1.000 mL of a 1.333 mmol L^–1^ KIO_3_ standard solution was transferred to a 250 mL Erlenmeyer flask. Subsequently, 1.0 mL of 3.0 mol L^–1^ H_2_SO_4_ and 5 mL of a 2.0% (w/v) KI solution were added. The resulting solution was titrated with 5 mmol L^–1^ Na_2_S_2_O_3_ solution until a pale-yellow color was observed, at which point the titration was briefly paused. At this point, five drops of a 1% (w/v) starch indicator solution were added, causing the solution to turn blue. The titration was resumed and continued until the blue color disappeared. For the standardization of the 20 mmol L^–1^ Na_2_S_2_O_3_ solution, a larger volume (4.000 mL) of the 1.333 mmol L^–1^ KIO_3_ standard solution and 2.0 mL of 3.0 mol L^–1^ H_2_SO_4_ were used, following the same titration procedure. All standardizations were performed in quadruplicate.

#### 2.2.2 Proposed Method for the Determination of Free Glycerol

An aliquot of 200 μL of biodiesel was weighed directly into a 10 mL screw-cap test tube. Subsequently, 1.0 mL of ethanol and 1.0 mL of a 10 mmol L^–1^ NaIO_4_ solution—both preheated to 50 °C —were added, and the mixture was vortexed vigorously for 3 min to complete the Reaction R1. The contents were then quantitatively transferred to a 250 mL conical flask, with the inner walls of the test tube rinsed using three portions of a 2.8 mol L^–1^ acetic acid solution (pH 3.0), totaling 10 mL of rinsing solution. After, 2.0 mL of a 0.30 mol L^–1^ Na_2_MoO_4_·2H_2_O solution was added, and the flask was shaken until a pale-yellow color appeared, indicating the formation of the [I­(MoO_4_)_6_]^5–^ complex (Reaction R2). Following this, 2.0 mL of a 2.0% (w/v) KI solution was added to initiate Reaction R3. The flask was then shaken vigorously and left to rest for 2 min. The resulting mixture was titrated with a 5 mmol L^–1^ Na_2_S_2_O_3_ solution, according to Reaction R4, until a light-yellow color was observed. At this point, five drops of a 1% (w/v) starch indicator solution were added, and the titration was resumed until the blue color disappeared for at least 30 s. Blank analyses were performed by replacing the biodiesel with deionized water.

Reaction 3 $${{\mathrm{IO}}_{3}}^{-}+8I^{-}+6H^{+}\to 3{I_{3}}^{-}+3H_{2}O$$

$$2S_{2}{O_{3}}^{2-}+{I_{3}}^{-}\to 3I^{-}+S_{4}{O_{6}}^{2-}$$ Reaction 4

Free glycerol content was calculated according to eq 5

1 $$\mathrm{GL}=\frac{C_{{\mathrm{Na}}_{2}S_{2}O_{3}}\times \left(V_{{\mathrm{Na}}_{2}S_{2}O_{3}}-V_{b}\right)\times 92.09}{m_{\mathrm{BD}}\times 12}\times 0.1$$

where: GL is the glycerol content, expressed as % (w/w); where: GL is the glycerol content, expressed as % (w/w); C_Na_2_S_2_O_3__ is the concentration of standard sodium thiosulfate solution, in mmol L; V^–1^; V_Na_2_S_2_O_3__ and Vb are the volumes (in mL) of Na_2_S_2_O_3_ used in the titration of the sample and the blank, respectively; 92.09 is the molar mass of glycerol, in g mol^–1^; *m*_BD_ is the mass of biodiesel analyzed, in mg; 12 is the stoichiometric factor; and 0.1 is a conversion factor used to express the final result as percentage by weight (% w/w).

#### 2.2.3 Proposed Method for the Determination of Total Glycerol

An aliquot of 100 μL of biodiesel was weighed directly into a screw-cap test tube, followed by the addition of 1 mL of 5% (w/v) KOH solution to promote saponification. The tube was shaken and then heated in a water bath at 95 °C for 10 min, with occasional agitation. After the reaction, the mixture was immediately neutralized with 0.5 mL of 12% (v/v) acetic acid solution, adjusting the pH to approximately 5. Subsequently, 1.0 mL of ethanol and 1 mL of 50 mmol L^–1^ NaIO_4_ solution (preheated to 50 °C) were added (Reaction R1). The mixture was vortexed for 3 min and then quantitatively transferred to a 250 mL Erlenmeyer flask, with the inner walls of the test tube rinsed using three portions of a 2.8 mol L^–1^ acetic acid solution (pH 3.0), totaling 10 mL of rinsing solution. After, 2.0 mL of a 0.30 mol L^–1^ Na_2_MoO_4_·2H_2_O solution was added, and the flask was shaken until a pale-yellow color appeared, indicating the formation of the [I­(MoO_4_)_6_]^5–^ complex (Reaction R2). Following this, 2.0 mL of a 2.0% (w/v) KI solution was added to initiate Reaction R3. The flask was then shaken vigorously and left to rest for 2 min. The resulting mixture was titrated with a 20 mmol L^–1^ Na_2_S_2_O_3_ solution until a light-yellow color was observed (Reaction R4). At this point, five drops of a 1% (w/v) starch indicator solution were added, and the titration was resumed until the blue color disappeared for at least 30 s. Blank analyses were performed by replacing the biodiesel with deionized water. All analyses were conducted in quadruplicate. Total glycerol content was calculated according to eq 5.

#### 2.2.4 Method Validation

The proposed methods for the determination of free and total glycerol in biodiesel were validated in terms of linearity, limits of detection (LoD) and quantification (LoQ), repeatability, reproducibility, and accuracy.

Linearity, LoD and LoQ were assessed by constructing a response curve using standard ethanolic solutions of glycerol within the ranges of 10.4–422 mg L^–1^ for free glycerol and 107–3144 mg L^–1^ for total glycerol. All measurements were carried out in triplicate by analyzing 1.00 mL of each standard solution. The LoD and LoQ values were calculated according to the standard deviation of 12 blank measurements (σ_b_) and the slope (*S*) of the corresponding response curve, using eqs 6 and 7

2 $$\mathrm{LoD}=\frac{3.3\times \sigma_{b}}{S}$$

3 $$\mathrm{LoQ}=\frac{10\times \sigma_{b}}{S}$$

Repeatability was evaluated by performing six replicate analyses of commercial, soybean, and palm kernel biodiesel samples under identical conditions on the same day. Reproducibility was assessed by repeating the analyses on three different days. Precision was expressed as the relative standard deviation (RSD, %), calculated for both intraday (repeatability) and interday (reproducibility) measurements.

Accuracy was assessed by means of recovery assays. For this purpose, commercial, soybean and palm kernel biodiesel were fortified with 100 μL of glycerol standard solutions at concentration levels ranging from 100 to 400 mg L^–1^ for free glycerol and from 1000 to 4000 mg L^–1^ for total glycerol. All assays were carried out in triplicate, and results were expressed as percentage recovery. Additionally, method accuracy was evaluated by comparing the results obtained with the proposed procedures to those achieved using the reference gas chromatographic method (ASTM D6584). Biodiesel samples 1 to 8 and commercial biodiesel were analyzed, and all measurements were performed in triplicate.

#### 2.2.5 Reference Method: ASTM D6584

The determination of free and total glycerol in biodiesel samples was carried out by gas chromatography with flame ionization detection (GC-FID), in accordance with the ASTM D6584 standard. Monoacylglycerols (MAG), diacylglycerols (DAG), triacylglycerols (TAG), and free glycerol analyses by GC were conducted in a Shimadzu GC-2010 gas chromatograph with the following temperature programming: 50 °C for 1 min, a ramp of 15 °C/min to 180 °C, a ramp of 7 °C/min to 230 °C, a ramp of 30 °C/min to 380 °C, held for 10 min. An Elite–5HT capillary column (PerkinElmer) was used (15 m x 0.32 mm inner diameter; 0.10 μm stationary phase). The carrier gas was helium at a constant 3 mL/min. A FID was used at 380 °C, using hydrogen at 45 mL/min and air at 450 mL/min; the washing solvent was *n*-heptane. The samples and standard solutions were diluted in pyridine, as per the standard method. In 2 mL flasks, approximately 100 mg sample or standard solution were weighed, and the mass was recorded. Then, 100 μL of the internal standards butanetriol and tricaprin and 100 μL MSTFA were added. The mixture was left to stand for 20 min at room temperature, after wich 8 mL *n*-heptane was added. Then the flask was sealed and shaken, and 1 μL of the derivatized sample was injected directly into the column using a cold on-column injector.

#### 2.2.6 Matrix Effect Evaluation

Since unsaturated fatty acids are susceptible to oxidation,^31^ it is reasonable to assume that they could react with periodate and potentially interfere with the analysis, especially at low glycerol concentrations. To evaluate possible matrix effects, a glycerol-free derivative from commercial biodiesel was prepared to simulate a blank. This matrix consisted predominantly of free fatty acids (FFA), obtained through complete saponification of the original sample. This biodiesel was selected due to its production from a wide variety of highly unsaturated feedstocks, including soybean oil, used frying oil, and animal fats.^31–33^

To obtain the glycerol-free matrix, 30 mL of biodiesel was saponified with 20 mL of a 50% (w/v) aqueous KOH solution. The mixture was heated for 20 min, with periodic additions of water to maintain adequate reaction conditions. After saponification, the mixture was acidified with 85% (w/w) H_3_PO_4_ to convert the soap into FFA. The resulting mixture was then washed five times with 60 mL portions of distilled water to remove residual glycerol and salts. After washing, phase separation, and drying at 80 °C for 2 h, 200 mg of the dried fatty acid phase was analyzed in six replicates using the procedure established for free glycerol determination.

#### 2.2.7 Statistical Analysis

The proposed methods were statistically compared with the reference ASTM D6584 method using paired *t* tests at a significance level (α) of 0.05. The null hypothesis assumed no difference between the methods (expected difference = 0). Additionally, method agreement was assessed using Bland–Altman plots,^34,35^ in which the differences between the methods were plotted against their respective means. The mean difference and the limits of agreement were calculated as mean ± t·SD (standard deviation), where t corresponds to the critical value of the Student’s t-distribution for *n* – 1 degrees of freedom and at α = 0.05.

## 3 Results and Discussion

### 3.1 Establishment of Reaction Conditions

The periodate-to-biodiesel molar ratio used in this study was adapted from the work of Bondioli and Della Bella, in which free glycerol was oxidized with periodate and subsequently quantified by the Hantzsch spectrophotometric reaction.^22^ The reaction time of 3 min for the complete oxidation of glycerol by periodate was established based on the study conducted by Hartman.^36^ The pH conditions and periodate-to-molybdate molar ratio adopted for periodate masking were based on the method described by Belcher and Townshend,^37^ originally applied to the titrimetric determination of iodate and periodate in aqueous matrices.

Ethanol was employed in the procedure to enhance the solubilization of biodiesel in the aqueous medium and facilitate the release of glycerol into the reaction phase. When 2.00 mL of a 10.0 mg L^–1^ glycerol standard solution prepared in ethanol was analyzed using the proposed procedure for free glycerol determination, a relative error of −64.8% was obtained, indicating incomplete oxidation. However, when both the standard solution and the periodate solution were individually preheated to 50 °C before being combined, complete oxidation was achieved, and the error decreased to 1.3%. Likewise, analyzing 1.00 mL of the same standard solution under the same preheating conditions yielded a relative error of only 0.4%. Based on these results, all subsequent analyses were performed using 1.00 mL of ethanol, with both reagents preheated to 50 °C prior to mixing.

The effect of saponification time was evaluated by treating soybean biodiesel with 5% w/v KOH at 95 °C for different durations, followed by glycerol quantification. Figure 1 shows that the total glycerol content increased during the initial minutes of saponification and remained relatively constant from 5 to 12.5 min. At 10 min, the value reached a stable maximum, indicating that this is an adequate time to ensure complete hydrolysis of the glycerides and the full release of bound glycerol.

**Figure 1:**
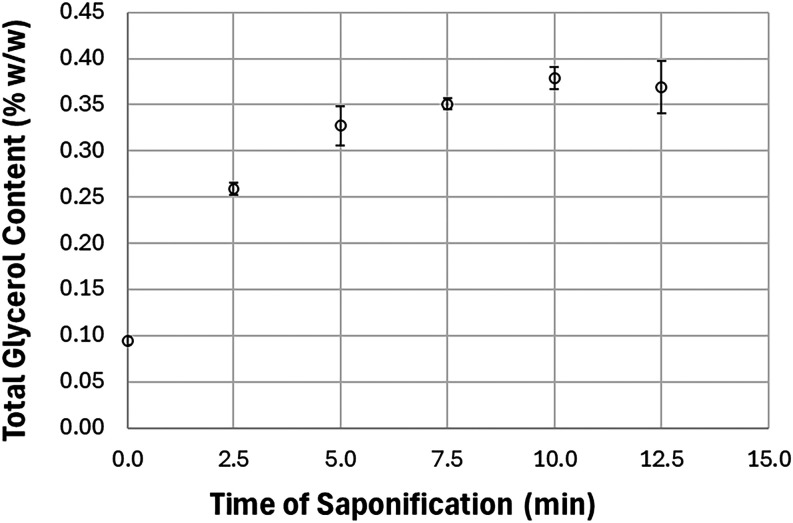
Effect of saponification time on total glycerol content in soybean biodiesel. Results represent the mean ± standard deviation from duplicate analysis.

### 3.2 Parameter Validation

#### 3.2.1 Response Curve Linearity

Response curves were constructed for glycerol by relating the titrant volume to the analyte concentration. As shown in Figure 2, both methods exhibited excellent linearity (*R* ^2^ > 0.99), in agreement with the criteria established by the AOAC validation guidelines.^38^

**Figure 2:**
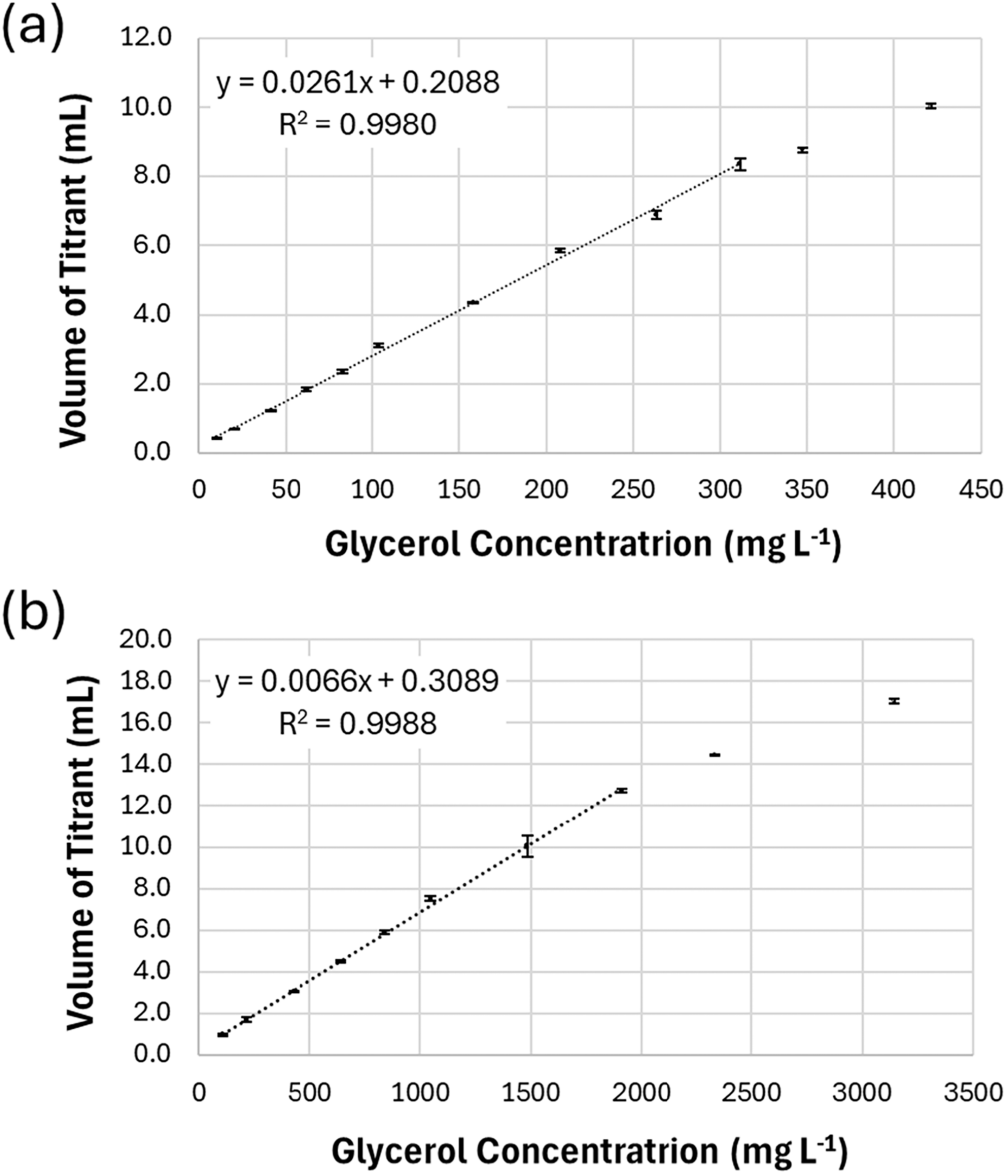
Linearity of the proposed titrimetric methods for the quantification of free (a) and total (b) glycerol using standard glycerol solutions. Data points outside the linear range and were not included in the regression. Results represent the mean ± standard deviation of three replicates.

The linear range obtained from the response curve for total glycerol determination (107 to 1911 mg L^–1^) was broader than that observed for free glycerol (10.4 to 312 mg L^–1^), mainly due to the higher concentration of sodium periodate used in the total glycerol method (50 mmol L^–1^ versus 10 mmol L^–1^). For concentrations above 312 mg L^–1^ (free glycerol) and 1911 mg L^–1^ (total glycerol), negative relative errors exceeding 10% were observed, likely due to kinetic limitations in the oxidation step, despite the use of periodate in stoichiometric excess.

#### 3.2.2 LoD and LoQ

The experimental results (Figure 2 and Table 1) indicated limits of detection of 1.42 mg L^–1^ for free glycerol and 5.35 mg L^–1^ for total glycerol. The corresponding limits of quantification were 4.32 and 16.2 mg L^–1^, respectively. Considering the biodiesel mass analyzed in the proposed methods, these values correspond to approximately 0.0016% (w/w) and 0.0059% (w/w) for the LoD, and 0.0048% (w/w) and 0.018% (w/w) for the LoQ, for free and total glycerol, respectively. These LoDs are about 12 and 30 times lower than the maximum levels established by regulatory agencies for biodiesel—0.02% (w/w) for free glycerol and 0.20% (w/w) for total glycerol—remaining below the regulatory thresholds and thus confirming the suitability of the proposed methods for monitoring these analytes in biodiesel samples.

**Table 1:** Blank, Sensitivity, And Limits of Detection/quantification Of The Proposed Methods

	free glycerol	total glycerol
blank ± σ_*b*_	0.131 ± 0.011 mL	0.130 ± 0.011 mL
sensitivity	0.0261 mL/mg L^–1^	0.0066 mL/mg L^–1^
LoD^a^	1.42 mg L^–1^ (0.0016% w/w)	5.35 mg L^–1^ (0.0059% w/w)
LoQ^a^	4.32 mg L^–1^ (0.0048% w/w)	16.2 mg L^–1^ (0.018% w/w)

a Values in parentheses correspond to the estimated % w/w limits, calculated based on the biodiesel mass analyzed in the proposed methods.

Additionally, the proposed method for free glycerol was approximately four times more sensitive than that for total glycerol (Table 1), which can be attributed to the use of a 4-fold more diluted titrant in the free glycerol procedure (5 mmol L^–1^ versus 20 mmol L^–1^).

The blank volumes obtained with the proposed methods were low and reproducible (Table 1), in stark contrast to those typically observed in the ABNT NBR 15771 and ABNT NBR 15344 methods, where blank titration volumes exceed those of the samples. In such cases, small fluctuations in the blank can lead to large relative errors, especially when the analytical signal (blank minus sample) is minimal. By effectively masking excess periodate with molybdate, the proposed procedures reduce blank consumption, ensuring a greater difference between sample and blank titration volumes. This leads to improved analytical accuracy and reliability, as well as enhanced resolution for detecting differences among samples with low glycerol content.

The ability of the proposed method to quantify such low glycerol levels can also be attributed to the low detection limit of triiodide when starch is used as an indicator (approximately 5 × 10^–7^ mol L^–1^),^39^ the high precision of the digital buret (±0.001 mL), and the use of a highly diluted titrant solution.

#### 3.2.3 Repeatability and Reproducibility

For the repeatability tests, analyses were performed six times on the same day, while reproducibility was assessed over three different days, totaling 18 measurements (Table 2). The RSD values for both free and total glycerol were consistently low, indicating satisfactory repeatability and reproducibility. In the case of free glycerol, RSD values for repeatability ranged from 2.4 to 8.8%, while for total glycerol, they varied between 3.3 and 7.2%. The RSD for reproducibility did not exceed 8.0%.

**Table 2:** Free and Total Glycerol Content (Mean ± Standard Deviation, % w/w) and Relative Standard Deviation (RSD, %) in Commercial, Soybean, and Palm Kernel Biodiesel Samples, Evaluated for Repeatability (*N* = 6) and Interday Reproducibility (*N* = 3)

biodiesel	day	free glycerol (% w/w)	RSD (%)	total glycerol (% w/w)	RSD (%)
Commercial	1	0.0075 ± 0.0004	5.6	0.184 ± 0.011	3.7
	2	0.0087 ± 0.0006	6.5	0.175 ± 0.012	7.2
	3	0.0079 ± 0.0002	2.4	0.227 ± 0.013	4.0
*Interday*		*0.0080 ± 0.0004*	5.2	*0.195 ± 0.016*	8.0
Soybean	1	0.0055 ± 0.0005	8.8	0.276 ± 0.010	3.7
	2	0.0053 ± 0.0004	8.3	0.278 ± 0.018	7.2
	3	0.0061 ± 0.0003	4.9	0.306 ± 0.012	6.4
*Interday*		*0.0056 ± 0.0004*	7.4	*0.287 ± 0.016*	5.8
Palm kernel	1	0.0272 ± 0.0013	4.9	0.597 ± 0.033	5.5
	2	0.0264 ± 0.0011	6.5	0.576 ± 0.019	3.3
	3	0.0273 ± 0.0009	3.1	0.613 ± 0.045	7.3
*Interday*		*0.0270 ± 0.0005*	1.8	*0.596 ± 0.019*	3.1

It was noted that the titration end point for biodiesel samples could initially be unstable in some cases. However, vigorous shaking of the conical flask for 30 s was generally sufficient to verify its stability and confirm whether the end point had been reached. According to ASTM D1615,^40^ iodometric titrations involving iodate and periodate ions may exhibit end point instability, as evidenced by the return of the blue color within 5 min in a resting flask. In such cases, the titration should be continued until the color change remains stable.

The three biodiesel samples investigated represent a broad spectrum of feedstock compositions: soybean biodiesel is rich in unsaturated fatty acid esters; palm kernel biodiesel consists predominantly of saturated esters; and commercial biodiesel is a heterogeneous industrial blend produced from vegetable oils, animal fats, and used cooking oil. Despite the inherent complexity and variability of the biodiesel matrix, the methods yielded consistent results across different samples and days, supporting their suitability for routine monitoring and preliminary screening purposes.

#### 3.2.4 Analyte Recovery

The recovery values obtained for free and total glycerol in commercial, soybean, and palm kernel biodiesel samples ranged from 90.9 to 106.0% (Table 3). According to the AOAC guidelines,^38^ acceptable recovery ranges are 90–108% for analyte concentrations between 0.1 and 1% (w/w), and 85–110% for concentrations within 0.01–0.1% (w/w). The recovery results demonstrate that the proposed methods provide accurate and reliable quantification of both free and total glycerol. The consistent recoveries observed across samples from diverse feedstocks and production routes highlight the robustness of the methods and their applicability to a broad range of commercial and laboratory-prepared biodiesel formulations.

**Table 3:** Recovery Test Performed by Adding Standard Solutions of Glycerol to Biodiesel^a^

biodiesel	spike (% w/w)	found (% w/w)	recovery (%)
*Free Glycerol*			
commercial	0.00555	0.00573	103.0
	0.01083	0.01086	100.3
	0.01666	0.01664	99.9
	0.02213	0.02182	98.6
soybean	0.00568	0.00587	103.4
	0.01144	0.01115	97.5
	0.01707	0.01591	93.2
	0.02235	0.02202	98.5
palm kernel	0.00572	0.00582	92.2
	0.01155	0.01218	105.4
	0.01737	0.01650	95.0
	0.02318	0.02206	95.2
*Total Glycerol*			
commercial	0.1053	0.1103	104.8
	0.2167	0.2298	106.0
	0.3219	0.3175	98.6
	0.4354	0.4172	95.8
soybean	0.1018	0.1039	101.8
	0.2063	0.2021	97.9
	0.3122	0.2836	90.9
	0.4163	0.3849	92.7
palm kernel	0.1042	0.1064	102.0
	0.2084	0.2110	101.3
	0.3146	0.3140	99.9
	0.4166	0.4219	101.7

a *N* = 3.

#### 3.2.5 Comparison with the ASTM D6584 Reference Method

To evaluate the accuracy of the proposed methods, biodiesel samples 1 to 8 and the commercial biodiesel sample were analyzed, and the glycerol contents were compared with those obtained using the reference method ASTM D6584. The results are broadly consistent between the reference and proposed methods, and the differences remain within an acceptable analytical range for screening and monitoring purposes (Table 4).

**Table 4:** Glycerides, Free Glycerol, and Total Glycerol Content (in % w/w) in Biodiesel Obtained by ASTM D6584 Standard and Titrimetric Proposed Method

	ASTM D6584 Standard (% w/w)					proposed methods (% w/w)	
samples	monoglycerides	diglycerides	triglycerides	free glycerol	total glycerol	free glycerol	total glycerol
1	0.184	0.018	0.015	0.020	0.237	–^a^	0.220 ± 0.005^c^
2	0.177	0.021	0.002	0.015	0.214	0.015 ± 0.002^b^	0.234 ± 0.003
3	0.166	0.017	0.002	0.012	0.198	0.011 ± 0.003^c^	0.222 ± 0.005
4	0.177	0.020	0.002	0.011	0.210	0.009 ± 0.001	0.203 ± 0.001
5	0.104	0.007	ND^d^	0.012	0.123	0.009 ± 0.001	0.126 ± 0.005
6	0.175	0.014	0.001	0.026	0.216	0.024 ± 0.003	0.232 ± 0.013
7	0.198	0.026	0.007	0.019	0.250	0.022 ± 0.001	0.268 ± 0.006
8	0.170	0.016	0.001	0.011	0.198	0.014 ± 0.001	0.267 ± 0.002
commercial	0.149	0.017	0.006	0.002	0.174	0.007 ± 0.002	0.184 ± 0.011

a Free glycerol content could not be determined by the proposed method due to limited sample volume;

b *N* = 6.

c *N* = 4.

d ND = not detected.

The paired *t* test was applied to evaluate the agreement between the results obtained using the proposed titrimetric method and those obtained using the reference ASTM D6584 method (Table 5). For free glycerol determination, no statistically significant difference was observed between the methods (*p* = 0.62), indicating strong consistency between them. In the case of total glycerol, a positive mean difference was observed, with the proposed method tending to provide slightly higher values than the reference method. However, the difference was not statistically significant at the 0.05 level (p = 0.098), suggesting acceptable agreement, albeit with a potential positive bias that warrants further investigation with a larger data set.

**Table 5:** Paired t-Test Comparing the Proposed Method and ASTM D6584 for Free and Total Glycerol Determination in Biodiesel

analyte	mean difference (% w/w)	standard deviation (% w/w)	number of paired observations	*t* (paired)	*p*-value	significant (*p* < 0.05)
free glycerol	0.0005	0.0029	8	0.52	0.62	no
total glycerol	0.015	0.024	9	1.87	0.098	no

Bland–Altman plots (Figure 3) showed that all data points were within the limits of agreement (mean ± *t*·SD), indicating consistency between the proposed and ASTM D6584 methods for both free and total glycerol. The absence of outliers suggests that the proposed method does not introduce systematic bias and performs reliably across the evaluated concentration range. Minimal bias was observed for free glycerol (0.0005% w/w), and although the method slightly overestimated total glycerol (0.015% w/w), the results remained consistent, supporting its suitability for routine analysis.

**Figure 3:**
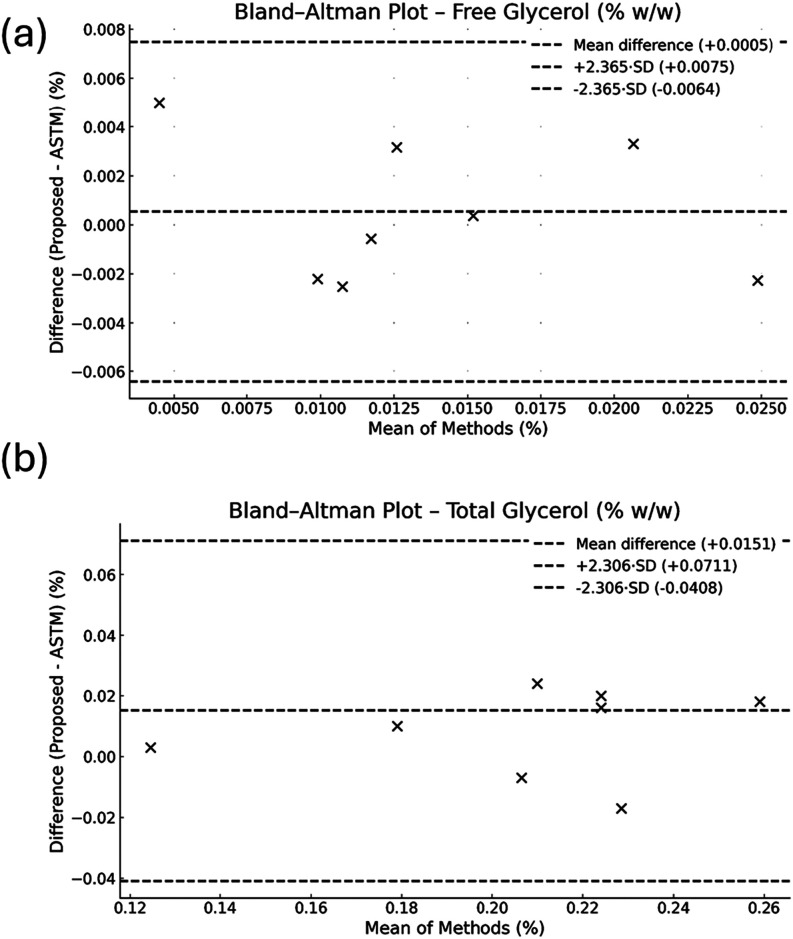
Bland–Altman plots comparing free glycerol (a) and total glycerol (b) content by the proposed methods and ASTM D6584. Dashed lines represent the mean difference and the 95% limits of agreement (±t·SD).

However, the free glycerol content determined by the proposed method for the commercial biodiesel was significantly higher than that obtained using the reference method (0.007% vs 0.002% w/w) (Table 4). This discrepancy may be associated with the presence of TBHQ, an antioxidant added by the manufacturer at a concentration of 120 mg L^–1^, according to the certificate of analysis. As a hydroquinone compound, TBHQ may undergo oxidation by periodate under the analytical conditions, potentially leading to an overestimation of glycerol content.^41^

The glycerol contents across the biodiesel samples (Tables 2–4) span values both below and above the limits established by regulatory agencies such as ANP, EPA, and CEN (0.02% w/w for free glycerol and 0.20–0.25% w/w for total glycerol). This distribution is particularly valuable, as it provides a basis for evaluating the method’s performance under both compliant and noncompliant conditions, reflecting a realistic scenario.

### 3.3 Matrix Effect

The analysis of the FFA sample derived from commercial biodiesel yielded an average value of 0.0019  ±  0.0005% (w/w), corresponding to approximately 10% of the regulatory limit for free glycerol (0.02% w/w) and about 1% of the limit for total glycerol (0.20–0.25% w/w). This concentration lies below the LoQ (4.32 mg L^–1^; ∼0.0048% w/w) and is close to the LoD (1.42 mg L^–1^; ∼0.0016% w/w), which constrains the reliability of the measurement at this level due to the method’s analytical sensitivity. Nonetheless, the result indicates a potential contribution from unsaturated fatty acid chains, which may be oxidized by periodate, leading to a slight overestimation of glycerol content. In practical terms, this suggests that the method may occasionally produce false positives (detecting glycerol levels slightly above regulatory thresholds when they are not) while the occurrence of false negatives is unlikely. Additionally, the presence of trace amounts of residual glycerol cannot be completely ruled out, despite the rigorous washing steps employed during sample preparation.

In summary, these findings suggest that, although minor matrix effects may be present, their influence is negligible under the tested conditions, particularly considering the strong agreement observed between the proposed and reference methods for free glycerol determination (Table 4) and in the recovery assays (Table 3).

### 3.4 Feasibility of Reference and Proposed Methods

The proposed methods offer a simpler, faster, and more effective approach for the determination of free and total glycerol in biodiesel when compared to ABNT NBR 15771 and ABNT NBR 15344 standards. Analyses can be completed in approximately 5 min for free glycerol and 15 min for total glycerol using the proposed methods, whereas the ABNT NBR standards require at least 40 and 70 min, respectively (Table 6).

**Table 6:** Methodological Comparison of Reference and Proposed Methods for Free and Total Glycerol Determination Per Biodiesel Sample

	free glycerol		total glycerol		free and total glycerol
methodological detail	proposed	ABNT NBR 15771	proposed	ABNT NBR 15344	ASTM D6584
principle	titrimetric	titrimetric	titrimetric	titrimetric	chromatographic
sample mass	200 mg	100 g	100 mg	10 g	100 mg
volumes of organic solvents	1 mL of ethanol	100 mL of diethyl ether	1 mL of ethanol	90 mL of chloroform and 100 mL of absolute ethanol	115 mL of pyridine^c^ and 8 mL of *n*-heptane
volume of glacial acetic acid	1.7 mL	25 mL	1.7 mL	25 mL	N/A^a^
analyte extraction time	N/A^a^	10 min	N/A^a^	10 min	N/A^a^
analyte modification time	3 min (oxidation)	30 min (oxidation)	13 min (saponification followed by oxidation)	60 min (saponification followed by oxidation)	20 min (derivatization with MSTFA^b^)
signal measurement time	2 min	2 min	2 min	2 min	32 min
total analysis time	5 min	42 min	15 min	72 min	52 min

a N/A = Not applicable (not required in the procedure).

b MSTFA = *N*-methyl-N-(trimethylsilyl)­trifluoroacetamide.

c For the preparation of the calibration curve.

Although the analysis time for a single sample is short, the analytical throughput is even more favorable. For free glycerol determination, it was possible to analyze three samples within 10 min, on average, since the oxidation step with periodate was carried out simultaneously in triplicate using a vortex mixer. Under these conditions, the analytical frequency reached 18 h^–1^, which is comparable to that of automated methods reported in the literature based on periodate oxidation of glycerol followed by spectrophotometric detection of formaldehyde via the Malaprade reaction (Reaction R1), which achieve frequencies of 14–16 h^–1^.^21,42^

The proposed procedures eliminate the need for successive analyte extractions and reanalysis, as recommended by ABNT NBR method, since the periodate was effectively masked by the molybdate, increasing the difference between the volumes used in the titration of the blank and the sample. In summary, they significantly streamline the analytical process by reducing both the time required and the operational complexity. Moreover, they effectively overcome the principal limitations inherent to the ABNT NBR methods, offering a more robust and practical alternative for the determination of free and total glycerol in biodiesel.

The chromatographic method (ASTM D6584) is widely recognized as the most reliable and accepted approach for the determination of free and total glycerol in biodiesel. It enables the simultaneous quantification of multiple analytes, including mono-, di-, and triglycerides, with high sensitivity and selectivity. Additionally, the method supports automated sample processing and requires the construction of an analytical calibration curve, which is particularly advantageous when analyzing large batches of samples. However, these benefits come with notable limitations, such as the high cost of instrumentation, the need for specialized personnel, and the use of expensive and chemically unstable standards. In contrast, titrimetric methods are more suitable for settings in which only a small number of samples are analyzed, offering a simpler and more cost-effective alternative for routine or preliminary assessments.

The proposed methods contribute to the principles of Green Chemistry, as they reduce or eliminate the need for toxic organic solvents such as pyridine, chloroform, ethyl ether, and *n*-heptane, which are required in the ASTM D6584, ABNT NBR 15344, and ABNT NBR 15771 standards (Table 6). These solvents pose significant health and safety risks to analysts and may be subject to purchase restrictions by security agencies in several countries, making their acquisition and handling more difficult. However, since the proposed methods are based on redox reactions, the presence of oxidizing or reducing impurities in the biodiesel samples may interfere with the analyses.

## Conclusions

The proposed titrimetric methods demonstrated excellent analytical performance for the determination of free and total glycerol in biodiesel. Both methods exhibited high linearity, in accordance with AOAC validation guidelines, with detection and quantification limits well below the maximum thresholds established by regulatory agencies such as ANP, EPA, and CEN. Repeatability and reproducibility were satisfactory, with low RSD values across different samples and days, and recovery tests yielded results within the acceptable range, even in samples derived from different feedstocks.

Comparison with the reference method (ASTM D6584) demonstrated statistically equivalent results for both free and total glycerol. Although minor matrix effects may be present, their influence was negligible under the tested conditions, particularly considering the strong agreement observed in both direct determinations and recovery studies.

The glycerol content observed in the analyzed biodiesel samples spanned values both below and above regulatory limits, allowing for a realistic assessment of method performance under compliant and noncompliant conditions. Despite the inherent complexity and variability of the biodiesel matrix, the methods showed consistent results, supporting their applicability for routine monitoring and preliminary screening.

The proposed methods are simpler, faster, and more economical than the reference chromatographic and titrimetric procedures. Moreover, they are consistent with certain aspects of Green Chemistry, as they reduce the reliance on toxic solvents such as chloroform, ethyl ether, pyridine, and *n*-heptane. Altogether, these characteristics make the proposed methods a practical and potentially more sustainable alternative for glycerol determination in biodiesel.

## Outlook

The detection and quantification limits of the proposed methods are close to critical regulatory thresholds. Therefore, these procedures are best positioned as complementary approaches, suitable for proof-of-concept applications, screening, or educational purposes, rather than full replacements for chromatographic methods in regulatory quality control, unless further sensitivity improvements are achieved. Moreover, the evaluation was performed on a limited set of biodiesel matrices, and additional studies addressing potential matrix effects—such as interferences from residual monoacylglycerols, soaps, or other cocontaminants—would be valuable to broaden their applicability. Finally, future work should also incorporate standardized green chemistry metrics to quantitatively substantiate the environmental claims.
